# *Passiflora incarnata L., herba,* in benzodiazepine tapering: long-term safety and efficacy in a real-world setting

**DOI:** 10.3389/fpsyt.2024.1471083

**Published:** 2024-10-04

**Authors:** Matteo Carminati, Mattia Tondello, Raffaella Zanardi

**Affiliations:** ^1^ Department of Clinical Neurosciences, Vita-Salute San Raffaele University, Milan, Italy; ^2^ Mood Disorder Unit, Istituto di Ricovero e Cura a Carattere Scientifico (IRCCS) San Raffaele Scientific Institute, Milan, Italy

**Keywords:** Passiflora, anxiety, depression, long-term treatment, benzodiazepine addiction, add-on treatment

## Abstract

**Introduction:**

Chronic and inappropriate benzodiazepine (BDZ) prescription and intake represent an important health and social concern worldwide. The aim of our study was to investigate the safety and efficacy of P. incarnata L., herba in reducing BDZ misuse in a real-world population of depressed and anxious patients in a long-term treatment with BDZs.

**Methods:**

Over an 18-month period (from July 2021 to December 2022), we previously conducted a retrospective and naturalistic study on euthymic outpatients with a diagnosis of anxiety or depression and chronically taking BDZs. In this study we contacted patients 12 months after their enrollment in the previous study to assess their disease status and their BDZs and P. incarnata intake.

**Results:**

Our findings support the effectiveness of a dry extract of P. incarnata L., herba, as an add-on treatment during BDZ tapering in patients with anxiety or depression. We confirmed this effect to be sustained over time, and P. incarnata showed to be easily discontinued with no rebound, withdrawal or psychological dependence effect. The absence of side effects and adverse events confirmed the safety of P. incarnata in a real-world population. Personality disorders confirmed to be a relevant risk factor for maintaining addictive behavior, even when symptoms associated to withdrawal seem to be not particularly relevant.

**Discussion:**

We confirmed the possible effectiveness of P. incarnata as an add-on treatment in BDZ reduction. Further studies may be helpful to better investigate the promising properties of P. incarnata in the management of relevant clinical issues, such as anxiety disorders and addiction, that are classically known to benefit from GABAergic treatments.

## 1 Introduction

Benzodiazepines (BDZs) have long been at the forefront of pharmacotherapy for various clinical conditions, ranging from anxiety disorders and mood disturbances to insomnia (1). Traditionally, the understanding of the pathophysiology underlying these conditions has been rooted in the monoaminergic theory, which emphasizes the role of neurotransmitters like serotonin, dopamine, and norepinephrine (2). Therefore, clinical guidelines agree on indicating serotoninergic agents as the proper pharmacological treatment for anxiety and depression. Nevertheless, due to their rapid effectiveness in reducing sleep disturbances and anxiety symptoms, BDZs are often the initial treatment prescribed by both general practitioners and other specialists (3, 4). However, concerns regarding their potential for abuse, development of dependence, and adverse effects on cognition and psychomotor function have raised among physicians in the last decades (5–8). These apprehensions are further compounded by the recognition of the substantial health and social issues globally associated with chronic and inappropriate BDZ use (9). The prevalence of BDZ prescriptions remains notably high, with rates ranging from 2% to 5% across different populations (10, 11).

The debate surrounding the long-term therapeutic utility of BDZs reflects the inherent complexities in their clinical management (12–15). While some evidence supports their efficacy in specific anxiety disorders, their widespread use often exceeds the scope recommended by current clinical guidelines (16–18).

Under a pharmacodynamical point of view, BDZs exert their therapeutic effects by enhancing GABAergic neurotransmission through positive allosteric modulation of GABAA receptors (19). This augmentation amplifies the inhibitory actions of GABA, resulting in sedative, anxiolytic, and hypnotic effects (19). However, it is important to recognize that these symptomatic improvements do not address the underlying neurobiological dysregulations associated with anxiety and mood disorders (20). Furthermore, prolonged BDZ use can induce neuroadaptive changes in reward circuitry, leading to cravings and withdrawal symptoms upon discontinuation, while also potentially causing multifaceted adverse effects that impact various dimensions of health and well-being (20, 21). In particular, chronic BDZ use is associated with impaired cognitive functioning, including decreased attention, memory, and executive function (21). These cognitive impairments can significantly impact daily functioning and quality of life. Prolonged BDZ exposure can exacerbate cognitive decline, particularly in older adults, further complicating the management of conditions like MDD and comorbid insomnia (21). Two additional factors linked to prolonged BDZ use, as observed in recent studies, are the insufficient amelioration of insomnia and the increased hospitalization rates among patients with MDD. This highlights the need for alternative treatments and careful monitoring to address the potential risks of long-term BDZ therapy (22). Moreover, recent meta-analyses suggested that prolonged BDZ use is associated with an increased risk of cancer and a higher risk of mortality (23, 24).

Discontinuing BDZ therapy poses relevant clinical issues due to the emergence of withdrawal symptoms, collectively termed the discontinuation syndrome. These symptoms range from rebound anxiety to specific abstinence manifestations such as headache, nausea, and muscular discomfort (25–28).

The phenomenon of BDZ misuse encompasses both intentional abuse and inadvertent dependency, each posing distinct challenges to healthcare providers. Intentional misuse may involve both polydrug abuse or the use of BDZs as the primary substance of choice, necessitating specialized interventions. In contrast, unintentional misuse ensues from prolonged therapeutic use, wherein patients inadvertently escalate dosages to alleviate withdrawal symptoms or sustain perceived benefits. The insidious nature of BDZ dependence often makes affected individuals unaware of their addictive behavior until attempts at discontinuation provoke withdrawal reactions (10).

Due to the clinical relevance of this issue, a new clinical concept has recently been proposed to better describe patients experiencing “significant physiological or functional decline during or after a BDZ taper”, defined as complex persistent benzodiazepine dependence (CPBD) (28). This new clinical category includes patients who rarely combine BDZ with not prescribed substances and who tend to take them as prescribed, with very low risk of overdose. On the other hand, they usually strongly rely on BDZ for their complete functioning, thus showing behaviors like early refills request or hoarding pills, especially during BDZ deprescribing (28).

The complexity of BDZ withdrawal necessitates tailored tapering strategies aimed at mitigating symptoms while ensuring patient safety (25, 29, 30). However, even when an appropriate tapering program is implemented, a subset of individuals may experience persistent symptoms, highlighting the need for adjunctive interventions (9). Recent research has explored the utility of non-benzodiazepine GABAergic agents in facilitating BDZ tapering and withdrawal management (31–34). Pregabalin and gabapentin, which modulate GABAergic neurotransmission via distinct mechanisms, have shown promise in ameliorating withdrawal symptoms (31–34). Additionally, various adjuvant medications have been investigated for their potential to augment BDZ tapering outcomes (35–45). While some agents, such as tricyclic antidepressants and anticonvulsants, have demonstrated efficacy in preliminary studies, others have yielded inconclusive or negative results (35–45).

Recently, a large interest has raised among clinicians regarding the use of second-generation antipsychotics for a range of non-psychotic conditions, including substance abuse and personality disorders. However, the off-label use of low-dose second-generation antipsychotics showed a weak correlation with cardiometabolic mortality, posing a relevant limit for their wide use among general population (46).

In light of the limitations of pharmacological interventions, attention has turned towards alternative modalities for managing anxiety and insomnia. Herbal remedies, such as *Passiflora incarnata*, have garnered interest due to their well-known anxiolytic and hypnotic properties (47–52). Indeed *P. incarnata*, commonly known as passionflower, has a rich history of traditional use in alleviating anxiety and promoting relaxation (48). Recent studies have elucidated its pharmacological mechanisms, revealing its modulation of the GABA system through interaction with GABAA and GABAB receptors (49–51). In a previous work by our group, we investigated the effectiveness of *P. incarnata L., herba*, as a tool in reducing BDZ misuse, facilitating the withdrawal regimen in a real-world population of depressed and anxious patients on treatment with BDZs. Our findings suggested the role of *P. incarnata* as an effective add-on treatment during BDZ tapering. Specifically, the better management of withdrawal symptoms and anxiety rebound allowed a faster reduction of BDZs in patients taking *P. incarnata* compared to patients undertaking a classical tapering program. We hypothesized a correlation between the known pharmacological effects on GABAA and GABAB receptors of flavonoid fraction of *P. incarnata* and the observed clinical outcome (52). One of the main limits of the previous study was the relatively short follow-up (3 months). Indeed, according to recent literature, among depressed patients, a large majority (up to 86%) starts again taking BDZs two years after a successful tapering program (53), and thus a long-term follow-up seems to be necessary to better assess the effectiveness of *P.incarnata* in enhancing the efficacy of BDZ tapering programs.

Aim of the present study was to confirm the possible role of *P. incarnata L., herba* as an add-on treatment for BDZ tapering, and its efficacy in maintaining BDZ discontinuation over time. We collected information of patients from our previous study after one-year follow-up. More specifically, we observed a more rapid reduction of BDZs, as compared to classical tapering, when using add-on therapy with *P. incarnata*. We confirmed this effect to be sustained over time, and *P. incarnata* showed to be easily discontinued with no rebound, withdrawal or psychological dependence effect. Moreover, we investigated possible risk factors for a poor outcome of BDZ tapering, highlighting a possible role of personality disorders as the most relevant clinical factors associated with a not complete BDZ discontinuation.

## 2 Materials and methods

Over an 18-month period (from July 2021 to December 2022), we previously conducted a retrospective and naturalistic study on euthymic outpatients with a diagnosis of anxiety or depression and chronically taking BDZs. Patients referred to outpatients facility of San Raffaele Hospital in Milan, Italy. In this study we contacted patients 12 months after their enrollment in the previous study to assess their disease status and their BDZ and *P. incarnata* intake.

### 2.1 Participants

93 patients were collected, undergoing BDZ reduction program and taking a dry extract of *Passiflora incarnata L., herba*, at a daily fixed dosage ranging from 200 mg to 600 mg, for a better control of anxiety and insomnia. Inclusion criteria were as follows: age >17 years; fulfilling the Diagnostic and Statistical Manual of Mental Disorders 5th edition (DSM-5) (54) for any of the featured anxiety or depressive disorders; clinical remission of the depressive episode (Hamilton Depression Rating Scale 21 items <17) (55); mild anxiety symptoms (Hamilton Anxiety Rating Scale ≤17) (56); chronic BDZ consumption (>6 weeks); and treatment with a SSRI, SNRI, or other antidepressants. Exclusion criteria were: a diagnosis of psychotic disorders, pharmacological treatment with antipsychotics or mood stabilizers, substance use disorder, any severe disease, pregnancy.

### 2.2 *Passiflora incarnata* L., herba

This drug has been approved in Italy by AIFA (Agenzia Italiana del FArmaco) as a medical product since November 2020 (Tractana^®^), it is available in 200 mg tablets, and it is commonly prescribed in our center for the management of mild anxiety symptoms and sleep disturbances, at increasing dose until effective starting from 200 mg or according to clinical experience (maximum recommended daily dosage of 1600 mg). No particular interaction with other drugs has been described. One tablet contains 200 mg of dry extract, equivalent to about 700-1000 mg of *Passiflora incarnata L., herba.* No specific information about pharmacokinetics and pharmacodynamics of the product is available since it is registered as a herbal product of traditional use. No patients had ever taken Tractana^®^ before the beginning of the observational period. The most common BDZ tapering schedule in our outpatient facility consists of a 25% reduction of dosage every 2 weeks and a slower taper of 12.5% every 2 weeks near the end of stopping. This program may be changed and tailored to the single patient based on specific clinical features.

### 2.3 Data collection

We collected sociodemographic and clinical data, including current treatments and diagnosis at baseline. BDZ dose was reported as diazepam milligram equivalents at each time-point (thereafter referred as mg-equiv). Anamnestic data were collected from patients’ medical records at baseline (T0), while BDZ assumption were collected at T0, after 1 month (T1), three months (T2), and 12 months (T3) from the start of BDZ reduction. The course of disease over time was investigated through patient interview and/or clinical records at T3. All clinical data were collected by a trained psychiatrist. The study, approved by the Ethical Committee of the Hospital, was conducted in accordance with the Declaration of Helsinki, and all patients’ data were treated confidentially and anonymously.

### 2.4 Statistical analyses

All statistical analyses were performed using JASP (57) (version 0.16.4) computer software; tables and graphs were obtained by JASP or Microsoft Excel (58) (version 15.59) software. All tests were two-tailed, with a statistical significance level set at <0.05. Continuous variables are expressed as mean ± standard deviation (SD), while categorical variables are reported as numerosity and percentages. Using the Shapiro–Wilk normality test, we verified the non-normal distribution of continuous variables in the global sample and both groups. Friedman’s non-parametric version of repeated measures ANOVA (rmANOVA) was performed on mean BDZ dosage, to assess the progression of the withdrawal regimen.

## 3 Results

The whole sample consisted of 93 patients diagnosed with depression or anxiety and undergoing a protocol of BDZ downtitration with the addition of a dry extract of *Passiflora Incarnata L., herba.* 6 patients dropped out because of moving to another city (3), change of hospital (1) or death (2). The sociodemographic and clinical features of the sample at baseline are reported in **Table 1**. All patients were taking one antidepressant during all the observation period, as well as one BDZ at baseline. The number of patients taking each molecule, the dose range and a list of other medications taken during the study can be found in **Supplementary Materials**. The range of BDZ dosage expressed in diazepam mg equivalent was 1.5 to 50 mg.

**Table 1:** Sociodemographics and clinical data of the sample.

Baseline demographic and anamnestic features	
Age (years, mean ± SD)	53.1 ± 15.6 (range 18-83)
Sex ratio (M/F)	26/61
Education (years, mean ± SD)	13.2 ± 3.51
Occupation (U/E)	13/74
Status (single/coupled)	21/66
Diagnosis (depression/anxiety)	41/46
Baseline clinical measurements	
HARS (mean ± SD)	6.58 ± 4.65
BAI (mean ± SD)	5.63 ± 4.42
HDRS (mean ± SD)	4.64 ± 3.68
BDI (mean ± SD)	4.82 ± 3.83

M, male; F, female; U, unemployed; E, employed; HARS, Hamilton Anxiety Rating Scale; BAI, Beck Anxiety Inventory; HDRS, Hamilton Depression Rating Scale; BDI, Beck Depression Inventory.

**Figure 1:**
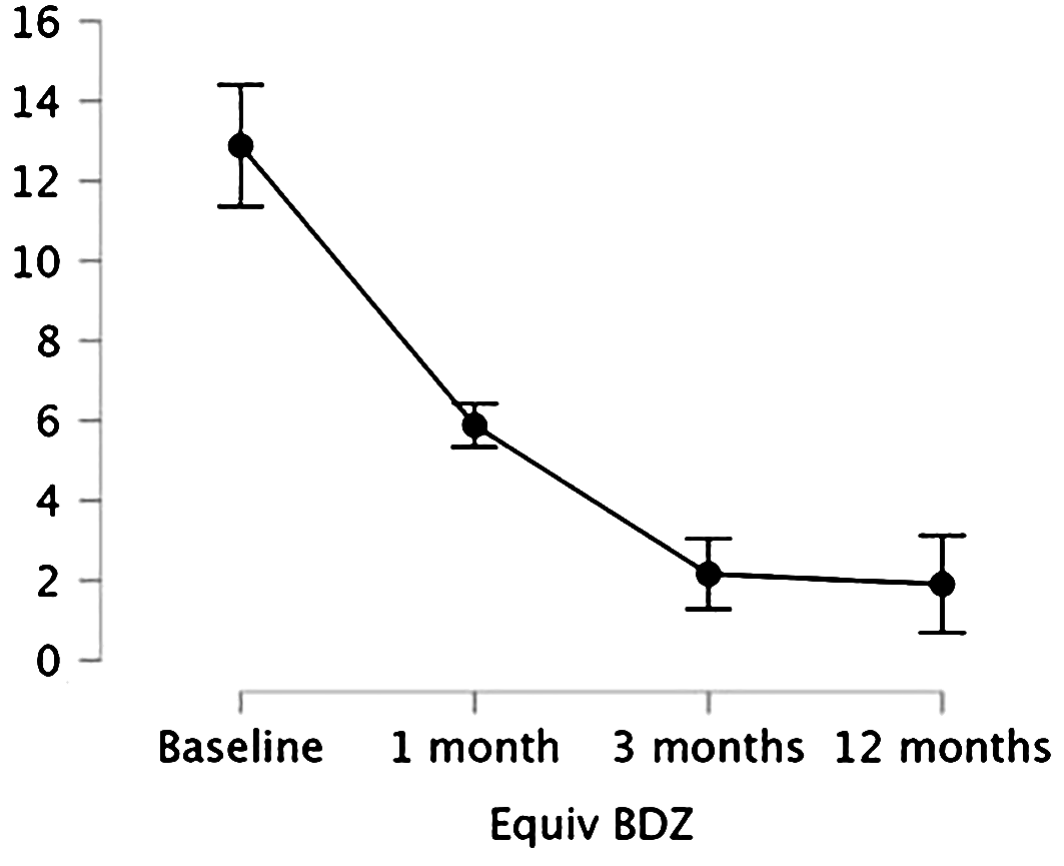
Means and 95% confidence intervals (vertical bars) of the BDZ dosage (mg-equiv) at baseline/pre-treatment (T0), at the end of the first month (T1), of the third month of treatment (T2), and after a 12-month follow-up (T3).

We performed a repeated-measures ANOVA to evaluate BDZ dosage (mg-equiv) change in the group over time, showing a significant effect of time (p < 0.001), meaning that BDZ dosage significantly changed over time. Holm’s *post hoc* comparisons confirmed that BDZ dosage showed a significant decrease within the group at each time point from baseline to 3 months (T0 vs. T1, mean difference: 3.47, p = 0.007; T0 vs. T2, mean difference: 10.91, p < 0.001; T1 vs. T2, mean difference: 3.81, p < 0.001), and the reduction was maintained at 12 months (T0 vs T3, mean difference: 10.98, p < 0.001; T1 vs T3, mean difference: 3.98, p < 0.001) while it showed no difference between 3 months and 12 months (T2 vs T3, mean difference: 0.253, p = 0.748). Mean values and standard deviations of BDZ mg equivalents over time are shown in **Figure 1**.

**Figure 2:**
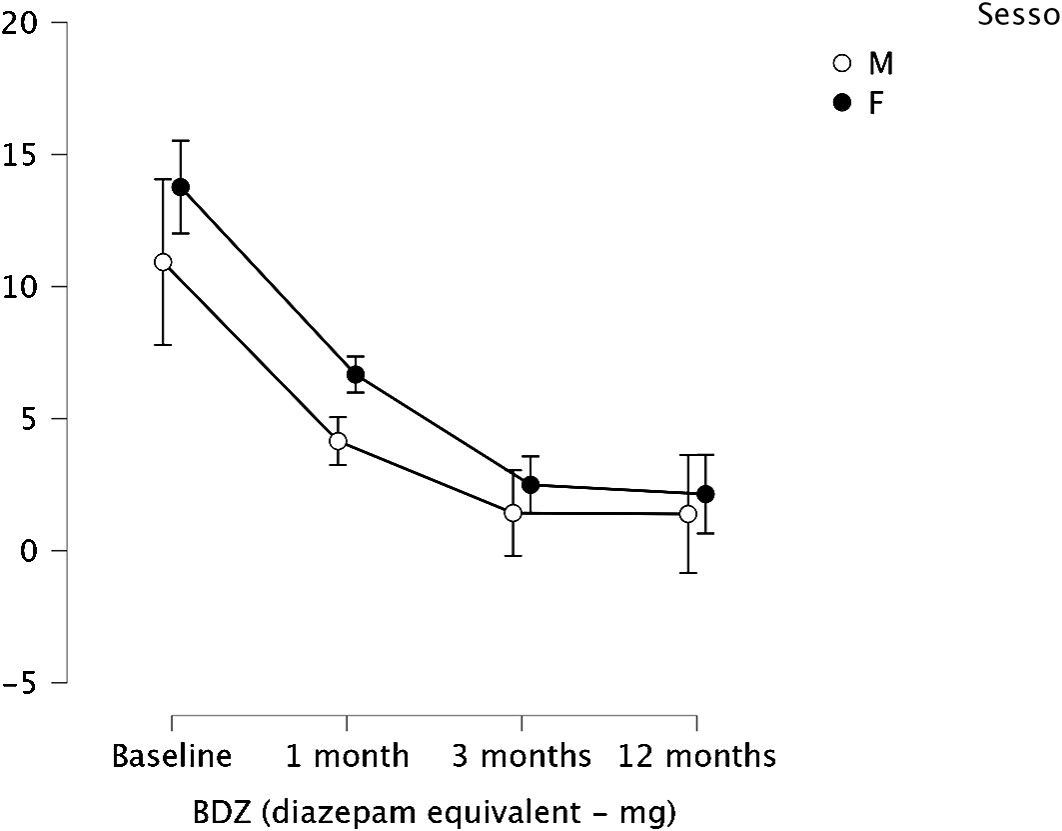
Means and 95% confidence intervals (vertical bars) of the BDZ dosage (mg-equiv) at baseline/pre- treatment (T0), at the end of the first month (T1), of the third month of treatment (T2), and after a 12-month follow-up (T3), split by sex (M, male; F, female).

Stratifying for sex, no difference emerged between male and female regarding BDZ dosage change over time (p = 0.526). The reduction of BDZ dose was confirmed considering the 2 groups separately, as shown in **Figure 2**.

After a 12-month follow-up, 69 out of 87 patients (79.3%) completely discontinued BDZ. Regarding relapsing of anxiety symptoms 5 out of 46 (10.87%) patients with anxiety showed a relapse with the need for a treatment change. 5 out of 41 (12.19%) of patients diagnosed with MDD showed a recurrence of disease during the 12- month observation. Only 3 out of 87 patients from the whole sample was still taking *P. incarnata* at the end of the 12-month follow-up, 2 at the dosage of 200 mg daily, one at 400 mg daily. We compared demographic and clinical features between patients who completely discontinued BDZ and patients who did not successfully discontinued BDZ. We did not found a statistically significant difference regarding age, sex, education, occupational status, marital status, smoke, alcohol use, as reported in detail in **Table 2**. We found a significant higher rate of personality disorder comorbidity in patients who did not discontinue BDZ compared to patients who completely discontinued BDZ (83.33% vs 28.99%, p value < 0.001).

**Table 2:** Sociodemographic and clinical data of the sample – comparison between groups.

	Complete BDZ discontinuation	Not complete BDZ discontinuation	*p value*
Age (years, mean ± SD)	52.77 ± 16.37	60.17 ± 16.47	0.108^a^
Sex (% female)	71.02%	61.11%	0.419^b^
Education (years, mean ± SD)	13.36 ± 3.62	13.61 ± 3.39	0.991^a^
Occupational status (% unemployed)	15.94%	5,55%	0.255^b^
Marital status (% coupled)	73.92%	77.77%	0.843^b^
Smoke	24.64%	22.22%	0.831^b^
Alcohol use disorder	5.79%	5.56%	0.969^b^
Personality disorder	28.99%	83.33%	< 0.001*^b^

SD, Standard Deviation; * *p* value < 0.05, statistical significance level; ^a^Mann-Whitney U-test; ^b^Chi-squared test.

## 4 Discussion

BDZs have indication for the symptomatic management of anxiety and sleep disturbances, conditions that should be properly addressed using other pharmacological or non-pharmacological therapies targeting the proper core of disease, as serotoninergic agents, psychotherapy or chronobiological strategies. The BDZ potential to induce addiction, that is one of the main reasons for reducing their use over time, is explained both with their great efficacy in rapidly reducing subjective symptoms of anxiety and insomnia, and to the development of withdrawal symptoms (28). Among these symptoms, a recurrence or a rebound of the ones that led to the initial BDZ prescription is possible, reinforcing the idea, in patients and caregivers, that BDZ treatment needs to be continued and prolonged over time to properly control the disease (35). This prolonged intake of BDZ enhances their well-known side effects and contributes to make more challenging a possible tapering program, both in terms of withdrawal syndrome severity and of motivation in reaching complete cessation (9). Therefore, both psychiatrists and general practitioners need effective tapering protocols, that may benefit from specific molecules targeting the GABAergic signaling, apart from the gradual individualized reduction of BDZ dose, in order to minimize withdrawal symptoms (59, 60).

Despite the clinical relevance of this issue, no specific strategies, both pharmacological or non-pharmacological, showed a clear effect in accelerating BDZ reduction to such an extent to be included in clinical guidelines for the management of BDZ addiction after a long-term use (11).

According to the existing literature about clinical effects of *P. incarnata L., herba*, both anxiety symptoms and craving or addiction may benefit from its pharmacological action. Since these symptoms represent the psychopathological core of BDZ abuse, a medical product based on a dry extract of *P. incarnata* may help in managing this condition (50).

Our results from a previous study appeared to confirm this hypothesis, showing a more rapid reduction, as compared to classical tapering, when using add-on therapy with *P. incarnata L., herba*. In particular, the group treated with *P. incarnata* showed a significantly greater reduction of BDZ mean dosage after both 1 and 3 months from the beginning of the tapering program. This difference was more relevant at the first time point, suggesting an effect of *P. incarnata* as an accelerator of BDZ tapering. Moreover, the observed clinical effect seemed to be dose-dependent, confirming the putative role of the drug besides its placebo effect. During the observation period, anxiety and depression symptomatology scores did not significantly change in both patient groups (52). We hypothesized that the action of *P. incarnata* as a modulator of the GABA system may be accounting for this clinical effect (61, 62). In particular, the agonism of GABAA post-synaptic receptor could explain the anxiolytic effect, well known from the traditional use of the plant. On the other hand, antagonism of GABAB pre-synaptic receptor may be accounting for the efficacy in reducing craving for GABAergic drugs as BDZs, as observed in animal models (63).

These preliminary results suggested that *P. incarnata* would not induce psychological dependence in patients accustomed to taking BDZ (in fact, replacing the dependence on low doses of BDZ with a new dependence), but rather would play a short- to medium-term role in accompanying the withdrawal process, making it faster as it is better tolerated. However, this speculative hypothesis needed a longer observation period to be confirmed (52).

In the present study the acceleration effect on benzodiazepine reduction of *P. incarnata*, rather than a substitutional one, has been substantiated by clinical observation after one-year follow up. Remarkably, nearly all patients ceased *P. incarnata* intake within a year of observation, indicating its temporary role in symptom management. This highlights its potential as a short-term intervention to facilitate BDZ reduction without serving as a long-term substitute. This finding also confirm the very low (or absent) risk of *P. incarnata* for inducing tolerance mechanisms, leading to a need to dose increase to maintain the clinical effect.

We already discussed about the important advantage of *P. incarnata* extract compared to BDZs in terms of safety and side effects (including impairment of performance and cognition) (64), in particular in the context of BDZ discontinuation, since performance impairment is the main concern about long-term BDZ treatment (52). This seems to be confirmed in the present study, since after a long-term observation no patients showed side effects or adverse events. On the other hand, patients showed a low rate of relapses, comparable to the general population of patients, and they did not need to change the ongoing pharmacological treatment, suggesting a maintained good global functioning. We also did not observed any side effect related to a reduction of efficacy of the current therapy taken by our patients, including a variety of drugs for cardiovascular, metabolic and endocrinological common diseases (see **Supplementary Material** for more details). Although no specific information is available about drud-drug interaction and the impact of *P. Incarnata* on P450 cytochrome, this clinical observation may suggest a low risk for relevant interaction with common use medications.

In our previous study on this sample of patients, we discussed the possible role of placebo effect in determining the observed differences between the groups. The fact no to interrupt any drug assumption may indeed have helped patients in maintaining well-being, also because patients who previously tried to stop taking BDZs may have experienced a rebound of symptoms, leading to a fear of drug discontinuation. Moreover, many patients may associate the ritual of taking pills with a positive healing effect. We also hypothesized that the EMA approval of a dry extract of *P. incarnata L., herba*, as a medical product could enhance this effect, since some patients who are used to take antidepressants in psychiatric settings may not trust the beneficial effects of herbal products or nutraceuticals, preferring a prescription of molecules with stronger preclinical and clinical evidence of efficacy. However, in this work we observed that the large majority of patients discontinued *P. incarnata* after a year of follow-up. Although an initial placebo effect may not be isolated given the design of the study, we can affirm that the effect was not responsible for the maintenance of abstinence. These data confirm that, even though a placebo effect was present, this may also have represented a useful tool in managing BDZ tapering in a subgroup of patients, not leading to the development of a new psychological addictive habit involving *P. incarnata* instead of BDZs.

In our sample very few patients did not completely discontinue BDZs, confirming the effectiveness of our tapering scheme program. On the other hand, identifying specific clinical features of this group of patients may help to detect possible risk factors for BDZ dependence and for a more difficult to manage BDZ tapering. As far as we could observe in our sample, we did not find specific risk factors for not completing BDZ discontinuation with the exception of personality disorder comorbidity. Personality disorder comorbidity is known to be a major risk factor for substance use disorders and addictive behavior, particularly in cluster B personality disorder, characterized by poor impulse control and emotional dysregulation. This subgroup of patients may benefit from a specific psychotherapy aimed to the management of addictive behavior. Indeed, the low dosage of BDZs taken by our patients also in this group may suggest that the difficulty in interrupting BDZ assumption is linked more to a psychological habit rather than biological dependence. Anyways, *P. incarnata* did not show any potential to induce addictive behavior even in this higher risk population. This sub-population is known to be at high risk for loss to follow-up in similar studies and for a relapse of abuse/misuse behavior. That could not be detected by the present study, and should be investigated by a longer observation.

To our knowledge, this is the first study to evaluate the long-term effectiveness of *P. incarnata L., herba*, in reducing BDZ misuse in people living with anxiety and depressive disorders. The non-randomized nature of the study offers a representative picture of the real-world situation, also considering the numerical consistency of the sample.

### 4.1 Limitations and strengths

The study has some limitations. The first is the retrospective and observational nature of this study, which did not include a placebo-controlled group. In line with previous studies and reviews on the topic, we used different outcome measures to evaluate the effectiveness of BDZ reduction, including the rate of complete BDZ cessation; mean dose reduction; rebound of anxiety and demoralization symptoms, as measured by clinical rating scales; and adverse events, defined as any undesirable medical event experienced by patients and collected from medical records (31). However, no craving rating scale was administered. Given the retrospective nature of the study, we could assess the compliance to the treatment only by asking patients themselves and caregivers. We also did not collect any blood sample, so we could not assess liver function or other biomarkers of safety of the drug in this specific population of patients, neither we had a specific measure of possible drug-drug interactions.

On the other hand, one of the main flaws of our previous study on the topic was the short follow-up period (3 months), that did not allow us to draw any firm conclusion regarding the sustained effect on BDZ interruption. This study did target this limitation investigating the long-term maintenance of the observed efficacy of *P. incarnata* in supporting BDZ tapering. In this longer period of observation, we could observe and measure relapse or dropout rates, which did not show in the previous study. No significant withdrawal effects were recorded.

Our work has important strengths. The most relevant is its novelty, since only a few studies were conducted on the clinical effect of *P. incarnata* on large population samples. Moreover, the observational nature of the study leads to a narrowing of the gap with clinical reality, giving information about the real-world feasibility of the treatment.

### 4.2 Conclusions and future directions

Our findings support the effectiveness of a dry extract of *P. incarnata L., herba*, as an add-on treatment during BDZ tapering in patients with anxiety or depression. More specifically, we had already observed a more rapid reduction of BDZs, as compared to classical tapering, when using add-on therapy with *P. incarnata*. We confirmed this effect to be sustained over time, and *P. incarnata* showed to be easily discontinued with no rebound, withdrawal or psychological dependence effect. The use of *P. incarnata* did not modify the usual disease course in patients with anxiety and depression, showing similar relapse rates compared with general population of patients. The absence of side effects and adverse events confirmed the safety of *P. incarnata* in a real-world population. Personality disorders confirmed to be a relevant risk factor for maintaining addictive behavior, even when symptoms associated to withdrawal seem to be not particularly relevant. These findings need to be confirmed by specifically designed clinical trials. Further studies may be helpful to better investigate the promising properties of *P. incarnata* in the management of relevant clinical issues, such as anxiety disorders and addiction, that are classically known to benefit from GABAergic treatments.

## Data Availability

The raw data supporting the conclusions of this article will be made available by the authors, without undue reservation.

## Supplementary material

Supplementary Materials List of antidepressants taken during the whole observation period. List of benzodiazepines taken at baseline. List of other medication taken by patients: beta-blockers (atenolol, bisoprolol); ramipril; olmesartan, valsartan; diuretics (furosemide, hydrochlorothiazide); levothyroxine; metformin; statins; acetylsalicylic acid.
