# Supplementary material for: Passiflora incarnata L., herba, in benzodiazepine tapering: long-term safety and efficacy in a real-world setting
Source: Front Psychiatry. 2024 Oct 4;15:1471083. doi: 10.3389/fpsyt.2024.1471083 (PMC11486640; doi:10.3389/fpsyt.2024.1471083)
Supplement: Supplementary Materials — List of antidepressants taken during the whole observation period. List of benzodiazepines taken at baseline. List of other medication taken by patients: beta-blockers (atenolol, bisoprolol); ramipril; olmesartan, valsartan; diuretics (furosemide, hydrochlorothiazide); levothyroxine; metformin; statins; acetylsalicylic acid. [file Table1.docx]

**Supplementary materials**

List of antidepressants taken during the whole observation period

| **Antidepressants** | **Number of patients** | **Dose range (mg/day)** |
| --- | --- | --- |
| *SSRIs* | *62* |  |
| Citalopram | 17 | 10-20 |
| Escitalopram | 2 | 10 |
| Fluoxetine | 8 | 20-40 |
| Fluvoxamine | 9 | 100-200 |
| Paroxetine | 15 | 10-40 |
| Sertraline | 11 | 100-200 |
| *SNRIs* | *18* |  |
| Duloxetine | 6 | 30-60 |
| Venlafaxine | 12 | 75-150 |
| *Others* | *7* |  |
| Clomipramine | 2 | 50-75 |
| Vortioxetine | 5 | 10-20 |

| **Benzodiazepines** | **Number of patients** | **Dose range (mg/day)** |
| --- | --- | --- |
| Alprazolam | 22 | 0.25-1 |
| Clonazepam | 4 | 0.5-1.5 |
| Delorazepam | 26 | 0.5-3 |
| Diazepam | 5 | 5-20 |
| Flurazepam | 11 | 15-30 |
| Lorazepam | 10 | 1-4 |
| Lormetazepam | 8 | 1-2 |
| Prazepam | 1 | 10 |

List of benzodiazepines taken at baseline

List of other medication taken by patients: beta-blockers (atenolol, bisoprolol); ramipril; olmesartan, valsartan; diuretics (furosemide, hydrochlorothiazide); levothyroxine; metformin; statins; acetylsalicylic acid.
